# HuR Affects the Radiosensitivity of Esophageal Cancer by Regulating the EMT-Related Protein Snail

**DOI:** 10.3389/fonc.2022.883444

**Published:** 2022-05-19

**Authors:** Yan Hu, Qing Li, Ke Yi, Chi Yang, Qingjun Lei, Guanghui Wang, Qianyun Wang, Xiaohui Xu

**Affiliations:** ^1^ Central Laboratory, The First People’s Hospital of Taicang, Taicang Affiliated Hospital of Soochow University, Taicang, China; ^2^ Department of Gastroenterology, The First People’s Hospital of Taicang, Taicang Affiliated Hospital of Soochow University, Taicang, China; ^3^ Department of General Surgery, The First People’s Hospital of Taicang, Taicang Affiliated Hospital of Soochow University, Taicang, China; ^4^ School of Pharmacy, Soochow University, Suzhou, China; ^5^ Department of Thoracic Surgery, the Third Affiliated Hospital to Soochow University, Changzhou, China

**Keywords:** esophageal cancer, HuR, snail, EMT, radiosensitivity

## Abstract

**Purpose:**

We previously found that Hu antigen R (HuR) can regulate the proliferation and metastasis of esophageal cancer cells. This study aims to explore the effects of HuR on the radiosensitivity of esophageal cancer.

**Materials and Method:**

Analyses of CCK-8, colony formation assay, Western blot, immunofluorescence, flow cytometry, reactive oxygen species (ROS), and mitochondrial membrane potential were conducted to characterize the esophageal cancer cells. Nude mouse models were used to detect the effects of HuR in a combination of X-ray treatment on the subcutaneous xenografts of esophageal cancer. In addition, a luciferase assay was used to detect the direct interaction of HuR with Snail mRNA 3’-UTR.

**Results:**

The down-regulation of HuR combined with X-ray can significantly inhibit the proliferation and colony formation of esophageal cancer cells. Flow cytometry data showed that the down-regulation of HuR could induce a G1 phase cell cycle block in esophageal cancer cells, and aggravate X-ray-induced apoptosis, indicated by the increases of apoptosis-related proteins Bax, caspase-3 and caspase-9. Moreover, the down-regulation of HuR could significantly impair the mitochondrial membrane potential and increase the ROS production and DNA double-strand break marker γH2AX expression in esophageal cancer cells that were exposed to X-rays. *In vivo* data showed that the down-regulation of HuR combined with radiation significantly decreased the growth of subcutaneous xenograft tumors. Furthermore, HuR could interact with Snail. Up-regulation of Snail can reverse the EMT inhibitory effects caused by HuR down-regulation, and attenuate the tumor-inhibiting and radiosensitizing effects caused by HuR down-regulation.

**Conclusion:**

In summary, our data demonstrate that HuR effectively regulates the radiosensitivity of esophageal cancer, which may be achieved by stabilizing Snail. Thus, HuR/Snail axis is a potentially therapeutic target for the treatment of esophageal cancer.

## Introduction

Esophageal cancer ranks the seventh in incidence and the sixth in mortality among all tumors. Approximately 70% of cases are men, and the incidence is 2-3 times higher in men than in women (1). Most patients with esophageal cancer are locally advanced when they are diagnosed, with large lesions, severe invasion, and lymph node metastasis (2). Therefore, the prevention and treatment of esophageal cancer has become a major public health and medical health issue of common concern all over the world (3).

Radiotherapy is one of the main ways for esophageal cancer treatment, but 70%-80% of patients that receive radiotherapy fail due to tumor recurrence or distant metastasis in the radiation field (4). Large-scale Phase III prospective clinical studies have confirmed that definite radiotherapy and chemotherapy can be used as the standard treatment for these patients (5). However, for patients with the same stage and the same treatment plan, the efficacy of radiotherapy is still various. In addition, even if the patient achieves complete clinical remission after treatment, a large proportion of patients have still relapse and metastasis in the later stage. In addition to factors such as clinicopathological classification, irradiation technology and treatment plan, the tumor heterogeneity caused by the difference in gene expression may have important roles in response to radiotherapy (6, 7). Thus, it is particularly important to find molecular biological indicators that can predict the efficacy of radiotherapy.

Epithelial-mesenchymal transition (EMT) is a process in which epithelial cells transform into mesenchymal cell morphology and acquire mesenchymal characteristics under specific physiological and/or pathological conditions. In this process, the cell polarity, the tight junctions and adhesion junctions between cells disappear, and the cells gain invasiveness and migration ability, which is important for tumor metastasis (8). Studies have shown that the occurrence of EMT in patients increases the resistance of tumor to radiotherapy and is closely related to tumor recurrence after radiotherapy (9, 10). Our previous study found that the down-regulation of HuR can significantly inhibit the expression of E-cadherin and promote the expression of MMP2, MMP9 and Vimentin (11). The expression level of E-cadherin is negatively correlated with metastatic potential in clinical tumors, and is designated as a tumor suppressor gene (12). Moreover, loss of E-cadherin promotes the radiation resistance of human tumor cells (13). In view of this, we explore how HuR regulates the radiosensitivity of esophageal cancer by regulating EMT.

In this study, we found that HuR can affect the radiosensitivity of esophageal cancer by regulating the proliferation, apoptosis and DNA double-strand breaks in esophageal cancer cells. And HuR can affect EMT by binding and regulating the expression of Snail. Snail participated in the regulation of radiosensitivity of esophageal cancer by regulating cell proliferation and apoptosis. Therefore, Snail may become a potential target for HuR-regulated radiosensitivity of esophageal cancer.

## Materials and Method

### Cells Culture, Transfection, Infection and Irradiation

Human esophageal cancer cell lines Eca-109, TE-1 and TE-10 were purchased from Cell Bank of Chinese Academy of Sciences (Shanghai, China). Eca-109 Cells were maintained in Dulbecco’s modified Eagle’s medium (Hyclone, Thermo Fisher Scientific, Waltham, MA, USA), while TE-1 and TE-10 was maintained in RPMI 1640 medium (Hyclone, Thermo Fisher Scientific, Waltham, MA, USA) supplemented with 10% fetal calf serum and 100 U/mL penicillin–streptomycin (37°C, 5% CO_2_).

The plasmid was used to up-regulate HuR and Snail, and the negative control (NC) plasmid was used as a control. For plasmid transfection, the cells were grown in a 6-well culture plate to 70-80% confluence and then transfected with 2.5 µg plasmids for 20 min using Lipofectamine 3000 (Invitrogen, Grand Island, NY, USA) at room temperature according to the manufacturer’s protocol. Forty-eight h after transfection, the cells were collected for further assays.

For viral infection, the shRNA targeting HuR lentivirus was obtained from Hanbio Biotechnology (containing RFP, Shanghai, China). The fragments were inserted into the lentivirus (LV) expression vectors LV-negetive control as control (shNC), LV-HuR sh1(shRNA-1, top strand, GCAGCATTGGTGAAGTTGAATCTGCATTCAAGAGATGCAGATTCAACTTCACCAATGCTGTTTTTT; bottom strand, AAAAAACAGCATTGGTGAAGTTGAATCTGCATCTCTTGAATGCAGATTCAACTTCACCAATGCTGC) and LV-HuR sh2(shRNA-2, top strand, GTACCAGTTTCAATGGTCATAATTCAAGAGATTATGACCATTGAAACTGGT ATTTTTT; bottom strand, AAAAAATACCAGTTTCAATGGCATAATCTCTTGAATTATGACCATTGAAACTGGTAC) (11) infected TE-1, Eca-109 and TE-10 cells. Seventy-two h after infection, the cells were subjected to a selection in medium containing 3 μg/mL puromycin (Sigma-Aldrich, St. Louis, MO) until all uninfected cells were killed with puromycin. Stable cell lines were validated using western blot.

TE-1, Eca-109 and TE-10 cells were irradiated with an X-ray linear accelerator (Rad Source Technologies) at a dose rate of 1.15 Gy/min.

### Cell Proliferation Assay

TE-1 and TE-10 cells infected with HuR shNC, sh1 and sh2 lentivirus or transfected up-regulating snail plasmid were resuspended to 1 × 10^4^/well in 100 μL in 96-well plates, with eight replicate wells for each set. After adherence, the cells were irradiated with 6 Gy of X-rays. Cell viability was measured at 0, 24, 48, and 72 h after irradiation using a CCK-8 kit (PF724, Dojindo Molecular Technologies, Kimamoto, Japan). A multi-functional microplate reader was used to detect the OD value at a wavelength of 450 nm. Graphpad 9.0.2 software (GraphPad Software Inc., San Diego, CA, USA) was used to analyze and plot the growth curve. Three independent experiments were performed in quadruplicates.

### Colony Formation Assay

The cells were seeded on 6-well plates at a density of 2000 cells/well. After 24 h, TE-1and Eca-109 were exposed to X-rays with a dose of 0, 2, 4, 6 and 8Gy. After 14 days of incubation at 37°C, the colonies were stained with Giemsa (418,033, Besso Biotechnology Co., Ltd., Zhuhai, China), and those with a minimum of 50 viable cells were counted. The cloning efficiency was calculated as a ratio of the number of colonies formed divided by the total number of cells plated.

### Flow Cytometric Analysis of Cell Apoptosis Assays

Following conventional digestion, cells in the logarithmic growth phase were used to prepare a single cell suspension; 2 × 10^5^ cells/mL were seeded in 6-well plates. 48h after X-ray irradiation, all cells were collected and centrifuged at 2000 rpm for 5 min. The supernatant was discarded after washing with PBS, and centrifugation was repeated twice. The cells were stained with fluorescein FITC-conjugated Annexin V and PI (40302ES60, Yeasen, Shanghai, China) and analyzed by flow cytometry (Beckman Coulter, Brea, CA, USA). The ratio of both early and late apoptosis cells was calculated.

### Flow Cytometric Analysis of Cell Cycle

48h after X-ray irradiation, cells in the logarithmic growth phase were collected and fixed with 70% precooled ethanol overnight. After staining with propidium iodide (10 µg/mL, Sigma-Aldrich, St Louis, MO, USA) in the dark for 30 min, flow cytometry was performed on a FACSCalibur Flow Cytometer system (BD Biosciences, San Jose, California, USA) and the cell cycle was analyzed with the Flow-Jo 7.6 software (BD Biosciences, San Jose, California, USA).

### DNA Double-Strand Break Assay and Immunofluorescence

DSBs represent an important ionizing radiation-induced lesion. The rapid phosphorylation of histone H2AX at serine 139 is a sensitive marker for DNA DSBs induced by ionizing radiation, which can later be detected by immunofluorescence.

The treated TE-1 cells were placed in 24−well plates with circular slides were fixed in 4% paraformaldehyde, permeabilized with 1% Triton X-100 for 10 min at room temperature, and then blocked with dilute 1% BSA (Solarbio, Beijing, China) for 1 h. The cells were incubated at 4°C overnight with the primary antibody γ-H2AX (Epitomics, Burlingame, CA, USA), which was diluted 1:1000 with 1% BSA. PBS was used to wash away the primary antibody. The cells were incubated with the secondary antibody at 37°C for 1 h, following which they were placed on coverslips and counterstained with 4-6-diamidino-2-phenylindole (Invitrogen) to mark the nuclei. Immunofluorescence staining was examined using a confocal microscope (Olympus Corporation) and then the mean number of γ-H2AX foci per cell (foci/cell) were counted.

### Measurement of Mitochondrial Membrane Potential

Esophageal cancer cells of different treatment groups were inoculated into a six-well plate at a cell density of 60%. The cells were irradiated with 6 Gy of X-rays. After 24 h, the medium was removed, and the cells were washed three times with PBS. Then, 200 µM of Mito-Tracker probe (1:1000, C1035, Beyotime, Shanghai, China) diluted in serum-free medium (RPMI1640) was added to each well. The cells were incubated at 37°C for 30 min. The medium was removed, the cells were washed three times with PBS and the cell nuclei were stained with Hoechst 33342 at a concentration of 5 μg/ml for 30 min at room temperature. The cells were visualized using a fluorescence microscope (DM IL LED, Leica, Wetzlar, Germany). The fluorescence intensity was then analyzed using ImageJ software (NIH Image for Macintosh, USA), and the ratio of Mito-Tracker and Hoechst was calculated.

### Detection of Oxygen Free Radicals

48h after X-ray irradiation, the cells were resuspended in a 96 well plate. The cells were incubated with ROS red working solution (ab186027, Abcam, Cambridge, UK) for 60 min at 37°C. Then they were irradiated with 6 Gy of X-rays. After 12 h, the cells were imaged at 20× under a microscope (DM IL LED, Leica, Wetzlar, Germany). The relative fluorescence of ROS in each cell was then statistically analyzed using ImageJ software.

### Western Blotting

The proteins in cell lysates were subjected to sodium dodecyl sulfate-polyacrylamide gel electrophoresis and transferred to a nitrocellulose membrane. After blocked with PBS/Tween-20 containing 5% bovine serum albumin, the membranes were incubated with following primary antibodies: HuR (1:1000, ab200342, Abcam, Cambridge, UK), Bcl-2 (1:500, ab692, Abcam, Cambridge, UK), cleaved Caspase-3 (1:500, ab2302, Abcam, Cambridge, UK), cleaved Caspase-9 (1:200, ab2324, Abcam, Cambridge, UK), Bax (1:1000, #5023, Cell Signaling Technology, Inc., Danvers, MA, USA),γ-H2Ax (1:1000, ab2893, Abcam, Cambridge, UK), Snail (1:1000, #3879, Cell Signaling Technology, MA, USA), matrix metalloproteinase 2 (MMP2) (1:1000, #40994, Cell Signaling Technology, Inc., Danvers, MA, USA), matrix metalloproteinase 9 (MMP9) (1:1000, ab38898, Abcam, Cambridge, UK), Vimentin (1:1000, #5741, Cell Signaling Technology, Inc., Danvers, MA, USA), E-cadherin (1:1000, #3195, Cell Signaling Technology, Inc., Danvers, MA, USA),β-actin (1:2000, GB1201, Servicebio, Woburn, MA, USA) and Tubulin (1:2000, ab176560, Abcam, Cambridge, UK). Goat anti-mouse primary antibodies (1:1000, A0208) or goat anti-rabbit secondary antibodies (1:1000, A0216) were from Beyotime (Shanghai, China). ImageJ software was used to analyze the relative expression of some proteins: the ratio of the gray value of the target protein to the reference protein.

### Luciferase Assay

A pmirGLO dual luciferase reporter containing the full-length 3’-UTR of Snail and two truncated Snail mRNA 3’- UTRs (ΔAREs, and AREs) were constructed by Gene Pharma (Shanghai, China) as reported (14). TE-1 and TE-10 cells were co-transfected with pmirGLO dual luciferase reporter with or without the constructions (full length, ΔAREs, AREs, or empty reporter) and HuR plasmid (or empty vector) using Lipofectamine 3000 reagent. The luciferase activity was measured with the Dual-Luciferase Reporter Assay System (Promega) 48h after transfection. The ratio of firefly luciferase to Renilla luciferase activities was used to determine promoter activities.

### Animal Experiments

Four-week-old male BALB/c nude mice were purchased from Shanghai SLAC Laboratory Animal Co., Ltd. (Shanghai, China). The mice were maintained under standard laboratory conditions on a 12-h light-dark cycle and given access to sterilized food and water under a specific pathogen-free environment. For the subcutaneous injection, TE-1 cells (5 × 10^4^) were suspended in 100 µL of PBS and inoculated subcutaneously into the right posterior flank region of the mice. The mice were divided into two groups: shNC group and sh1 group. Two-dimensional measurements were taken with an electronic caliper, and the tumor volume in cm^3^ was calculated using the formula: volume = a × b2/2, where a was the longest diameter and b the shortest diameter. On the fifth day after inoculation, the tumor *in situ* was irradiated with 4 Gy of X-rays for two consecutive days. When the subcutaneous tumor grows out, they were measured for two consecutive days, and then they were measured every 3 days, and the mice were sacrificed on the 21th day. Average of tumor weights for each group were calculated on the 21th day. The method of sacrifice of the animals was as follows: The nude mouse was placed into the euthanasia box, and carbon dioxide was infused into the box at a rate of 10–30% of the solvent in the euthanasia box per min. It was ensured that the nude mouse did not move, had no breathing and had dilated pupils. The carbon dioxide was turned off, followed by observation for 2 min to confirm that the nude mouse was dead.

### Statistical Analysis

Data are expressed as means ± standard deviation. The data were evaluated using an unpaired two-sided Student’s t-test after confirming that the data met appropriate assumptions (normality, homogeneous variance, and independent sampling). When more than two groups were compared, one-way ANOVA was adopted followed by Tukey’s *post hoc* test. For all *in vitro* experiments, three biological replicates were analyzed. For all *in vivo* experiments, five biological replicates were analyzed for each condition. Statistical analysis was performed using Prism 9.0.2 software (GraphPad Software, La Jolla, CA, USA). The differences were considered significant if *p* < 0.05 (*), *p* < 0.01 (**), *p* < 0.001 (***), and *p* < 0.0001 (****).

## Results

### HuR Downregulation Promotes the Effects of X-ray on the Proliferation and Cell Cycle of Esophageal Cancer Cells

**Figure 1:**
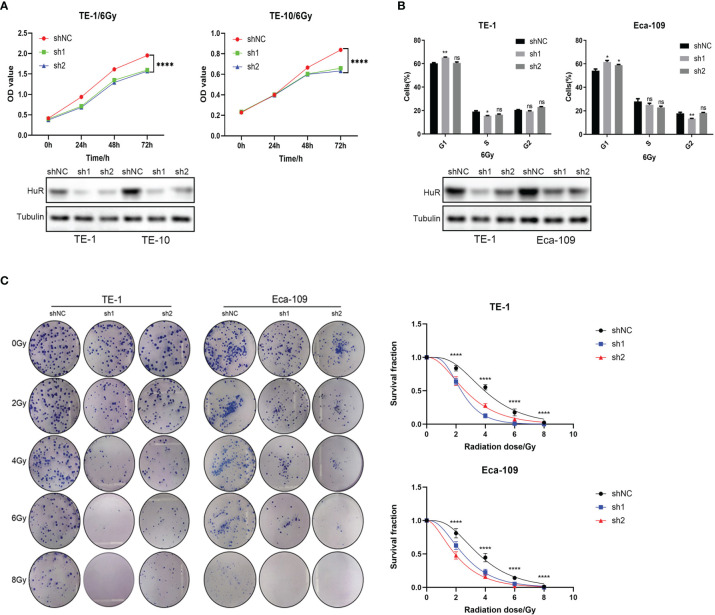
HuR was involved in the effect of X-ray on the proliferation of esophageal cancer cells. **(A)** CCK8 is used to detect the proliferation of esophageal cancer cells combined with X-ray after HuR downregulation. HuR downregulation inhibits the proliferation of esophageal cancer cells. **(B)** HuR downregulation increases the proportion of cells in G1. **(C)** The effect of HuR on the colony formation rate of esophageal cancer cells is verified by the colony formation test. The bar graph shows the colony formation rates. HuR downregulation decreases the colony formation of esophageal cancer cells. ns >0.05, *p < 0.05, **p < 0.01 and ****p < 0.0001.

Cell proliferation was monitored using CCK-8 after X-ray irradiation, showing that the down-regulation of HuR could significantly inhibit cell proliferation of esophageal cancer (**Figure 1A**). Flow cytometry analyses showed that the down-regulation of HuR obviously caused G1 phase arrest 48 h after X-ray irradiation (**Figure 1B**). Furthermore, the colony formation assay demonstrated that down-regulation of HuR significantly inhibited the clonogenicity of esophageal cancer cells after radiation (**Figure 1C**). Taken together, our data suggest that X-ray irradiation a significantly inhibits the proliferation and colony formation and increases the radiation sensitivity in esophageal cells that HuR is knocked down.

### HuR Downregulation Aggravates X-ray-Induced Apoptosis in Esophageal Cancer Cells

**Figure 2:**
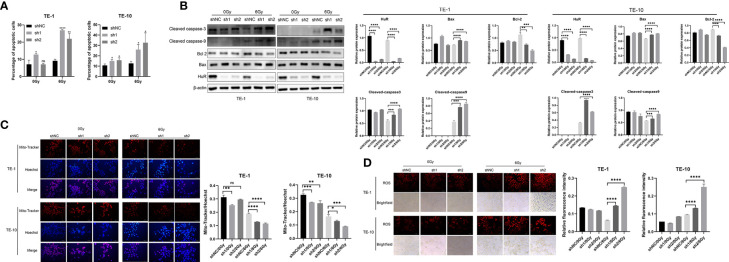
Effect of HuR on X-ray irradiation, cell apoptosis and reactive oxygen species and mitochondrial membrane potential of esophageal cancer cells. **(A)** Flow cytometry detection of the effect of HuR on the cell apoptosis of esophageal cancer cells. The bar graph shows the proportion of apoptotic cells. HuR downregulation increases cancer cell apoptosis. **(B)** Expression of apoptosis-related proteins Bax, Caspase-3, Caspase-9, and Bcl-2 determined by Western blot. **(C)** Mitochondrial membrane potential of esophageal cancer cells determined by Mito-Tracker immunofluorescence. **(D)** Immunofluorescence detection of the effect of HuR on reactive oxygen species (ROS) of esophageal cancer cells. ns > 0.05, *p < 0.05, **p < 0.01, ***p < 0.001 and ****p < 0.0001.

Flow cytometry was used to detect the apoptosis of esophageal cancer cells. It was shown that the down-regulation of HuR significantly promoted the apoptosis of esophageal cancer cells caused by X-rays (**Figure 2A**). In addition, the down-regulation of HuR significantly promoted the expression of pro-apoptotic proteins Bax, caspase-3, and caspase-9, and inhibited the expression of anti-apoptotic protein Bcl-2 (**Figure 2B**). However, in esophageal cancer cells with HuR up-regulated, X-ray-induced apoptosis was significantly inhibited (**Supplementary Figure 1**). Moreover, in esophageal cancer cells, X-rays induced more loss of mitochondrial membrane potential (**Figure 2C**) and promoted more generation of reactive oxygen species (**Figure 2D**) in HuR knockdown cells than in control cells. Thus data suggest that HuR downregulation significantly increases the apoptosis of esophageal cells induced by X-rays.

### HuR Downregulation Promotes DNA Damage of Esophageal Cancer Cells Caused by X-rays

**Figure 3:**
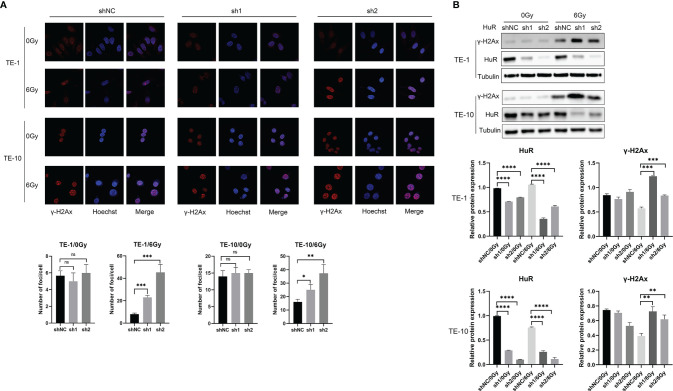
HuR downregulation increased γ-H2AX foci caused by X-ray irradiation and retarded DNA double strand breaks repair. **(A)** Immunofluorescence detection of γ-H2AX foci of esophageal cancer cells exposed to 6Gy X-ray irradiation after HuR downregulaiton. **(B)** Expression of γ-H2AX was determined by Western blot. ns >0.05, *p < 0.05, **p < 0.01, ***p < 0.001 and ****p < 0.001.

It is well known that γ-H2AX inducates DNA damage. In esophageal cancer cells that were exposed to X-rays, the number of γ-H2Ax foci were increased more in HuR dowregulation cells than in control cells (**Figure 3A**). Western blot data also showed that after irradiation, the level of γ-H2AX protein was significantly increased in cells that HuR was down-regulated (**Figure 3B**). These data suggest that HuR modulates the sensitization of esophageal cancer cells to irradiation by augmenting irradiation-induced DSBs.

### Down-Regulation of HuR Inhibits the Formation of Esophageal Cancer Subcutaneous Tumors

**Figure 4:**
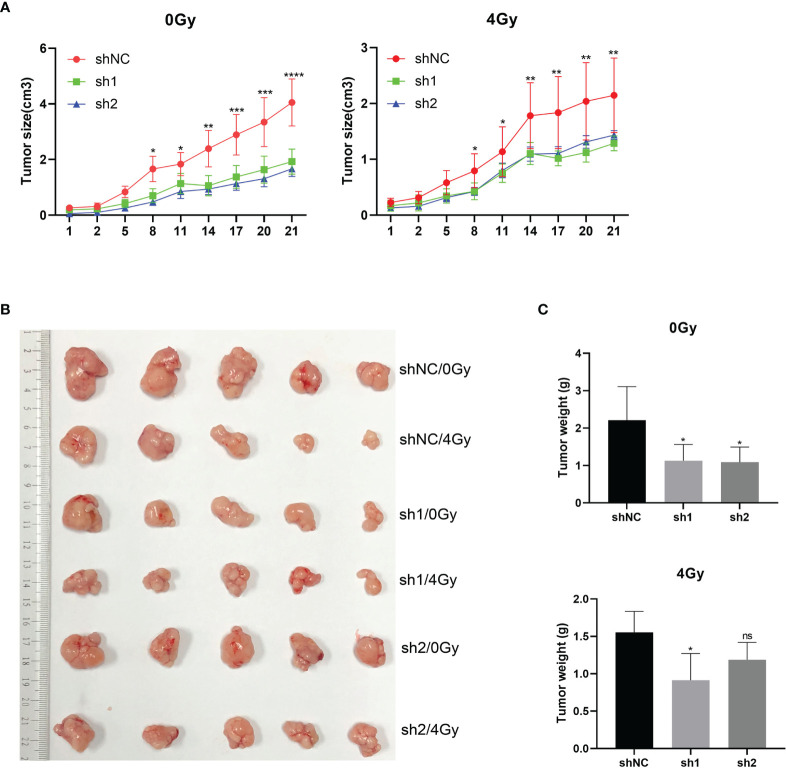
*In vivo* experiments supported the effect of esophageal cancer on X-ray radiosensitivity after HuR downregulation. Four-week-old BALB/c nude mice were divided into three groups: (1) shNC group; (2) sh1 group; and (3) sh2 group (n = 5/group). **(A)**Tumor volume growth curve of each group. Two-dimensional measurements were taken with an electronic caliper, and the tumor volume in cm3 was calculated. **(B)** Representative subcutaneous tumors of each group. **(C)** Average of tumor weights for each group. ns >0.05, *p < 0.05, **p < 0.01, ***p < 0.001 and ****p < 0.001.

After subcutaneously inoculating the esophageal cancer cells that were transfected with shNC and sh1 lentivirus into nude mice, the animals were exposed to 0Gy and 4Gy X-ray radiation for 48 hours. Tumor formation was significantly inhibited in mice that were inoculated with the cells that HuR was knocked down. What’s more, down-regulation of HuR combined with radiation strengthened the subcutaneous tumor suppressive effect of esophageal cancer (**Figures 4A–C**).

### HuR Interacts With 3’-UTR of Snail to Regulate Its Expression

**Figure 5:**
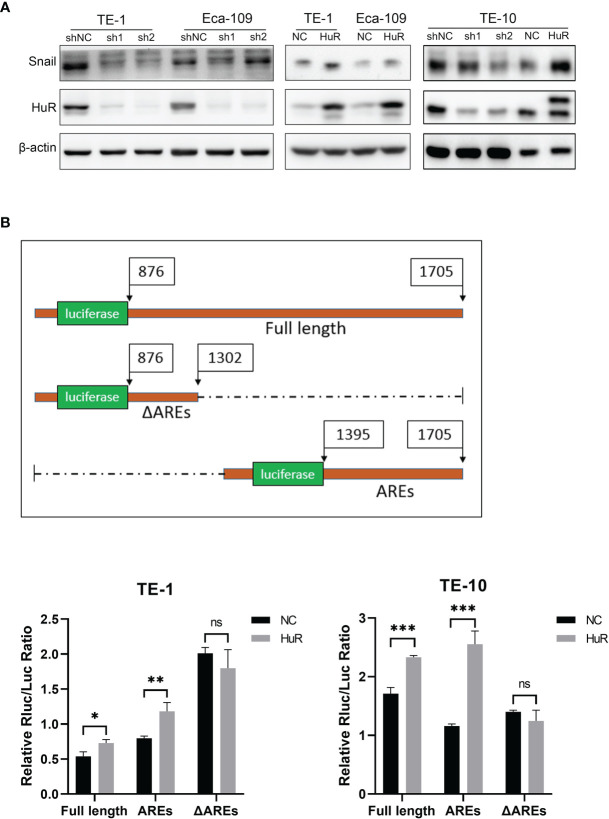
HuR positively regulated the expression of Snail in esophageal cancer cells. **(A)** Western blot was used to evaluate the expression of Snail in esophageal cancer cells in which HuR was downregulated or upregulated. **(B)** Schematic diagram of the constructions of the full length and 2 truncations of 3’-UTR of Snail mRNA into the dual-luciferase reporter. TE-1 and TE-10 cell lines were co-transfected with HuR (or vector) and the dual-luciferase reporter with Snail 3’-UTR constructions (either the Full length, AREs or ΔAREs, or empty reporter). After 48-hour incubation, luciferase activity was measured using the Dual-Luciferase Reporter Assay System (Promega). ns > 0.05, *p < 0.05, **p < 0.01 and ***p < 0.001.

In the cells that HuR was knocked down, we found that the expression of Snail was down-regulated. Coincidentally, the expression of Snail was significantly increased when HuR was up-regulated (**Figure 5A**). The direct interaction of HuR with Snail mRNA 3’-UTR was examined with a luciferase reporter assay. The full length 3’-UTR, ΔAREs and AREs which contain a firefly luciferase gene under the PGK promoter were each constructed into the pmirGLO vector. The sequence of ΔAREs did not contain the AU-rich HuR binding elements, and the sequence of AREs contained the major part of the AU-rich elements in the 3’-UTR.Luciferase assays showed that reporters only with full-length or ARE 3’UTR, HuR upregulation could enhance luminescence signal in both TE-1 and TE-10.However, the luminescence signal of reporter lacking HuR binding elements (ΔAREs) did not change with HuR upregulation (**Figure 5B**). In summary, these results indicated that the 3’-UTR of Snail harbors a binding site for HuR and that HuR regulates the expression of Snail protein in esophageal cancer cells.

### The EMT-Related Protein Snail Is Involved in the Radiation Tolerance of Esophageal Cancer Cells

**Figure 6:**
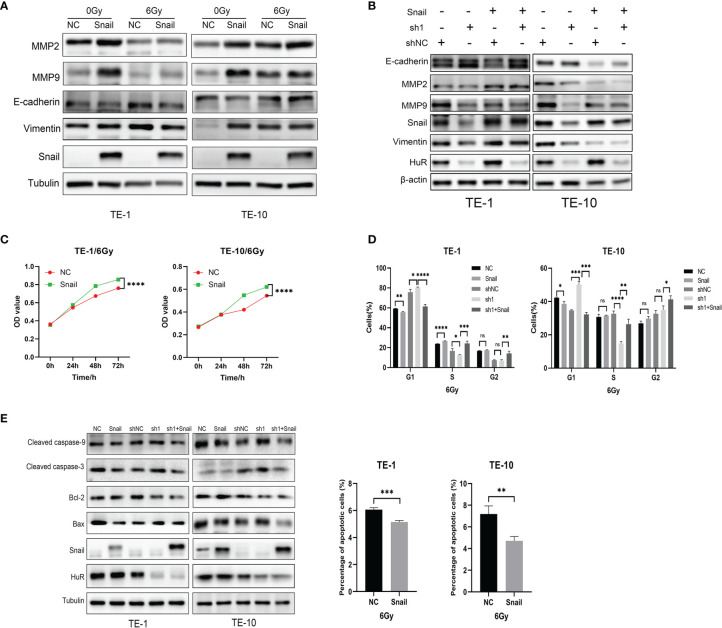
Snail attenuated the effects of X-rays on esophageal cancer cells. **(A)** Western blot was used to evaluate the expression of the expression of EMT-related proteins E-cadherin, MMP2, MMP9 and Vimentin in esophageal cancer cells after Snail was upregulated. **(B)** Western blot was performed to state that Snail can reverse the inhibitory effect of HuR on EMT-related proteins. **(C)** CCK8 was used to detect the proliferation of esophageal cancer cells combined with X-ray after Snail upregulation. **(D)** Flow cytometry detection of the effect of Snail on the cell cycle of esophageal cancer cells. The bar graph shows the proportion of cells. **(E)** Expression of apoptosis-related proteins Bax, Caspase-3, Caspase-9, and Bcl-2 were determined by Western blot. And Flow cytometry was used to detect the cell apoptosis of esophageal cancer cells after Snail upregulated. The bar graph shows the proportion of apoptotic cells. ns > 0.05, *p < 0.05, **p < 0.01, ***p < 0.001 and ****p < 0.001.

Western blot results showed that the up-regulation of Snail inhibited the expression of EMT-related E-cadherin, and promoted the expression of MMP2, MMP9 and Vimentin (**Figure 6A**). Moreover, up-regulation of Snail reverse the expression of these EMT-related proteins in HuR knockdown cells (**Figure 6B**). In addition, the up-regulation of Snail significantly reduces the effect of X-rays on the survival of esophageal cancer cells (**Figure 6C**). Furthermore, the up-regulation of Snail can significantly attenuate the G1 block of esophageal cancer cells caused by the down-regulation of HuR (**Figure 6D**).As well as up-regulation of Snail also significantly decreased the apoptosis rate of esophageal cancer cells caused by X-rays, and at the same time affected the expressions of apoptosis-related proteins in esophageal cancer (**Figure 6E**). Therefore, these results show that Snail is one of the important genes that HuR regulates the radiosensitivity of esophageal cancer.

## Discussion

Our previous studies reported that the expression of HuR in esophageal cancer tissues was significantly higher than that in adjacent tissues. Down-regulation of HuR can significantly inhibit the proliferation and metastasis of esophageal cancer cells. And, HuR can significantly promote the expression of EMT-related protein E-cadherin, and inhibit the expression of MMP2, MMP9 and Vimentin (11). Studies have found that HuR is involved in the regulation of radiotherapy of variety of tumors. Dou et al. found that LncRNA FAM83H-AS1 can affect the tolerance and cell metastasis of ovarian cancer by regulating the stability of HuR (15). Visvanathan et al. found that the recruitment of HuR to m6A-modified RNA is essential for SOX2 mRNA stabilization by METTL3 and that HuR preferentially binds to m6A-modified transcripts and participates in the radiation resistance of glioblastoma (16). Coincidentally, Yang et al. found that MiR-146b-5p can cause radioresistance of glioma by targeting HuR/lincRNAp21/β-catenin pathway (17). However, it is rarely reported that HuR is involved in the radiation therapy of esophageal cancer. In this study, we found that HuR affects the X-ray radiation sensitivity of esophageal cancer by regulating the EMT-related protein Snail. With X-ray irradiation, the proliferation, colony formation rate and mitochondrial membrane potential of esophageal cancer cells was significantly lower in HuR knockdown cells than in the control cells, while X-ray-induced apoptosis, DNA double-strand breaks, and ROS were significantly increased. In addition, animal experiments have also verified that esophageal cancer subcutaneous tumors grow more slowly in the presence of X-rays after HuR is down-regulated. HuR can regulate the expression of Snail, and the expression of snail is positively correlated with HuR. After HuR is down-regulated, the expression of EMT-related protein E-cadherin increased while the expression of MMP2, MMP9, and Vimentin decreased. However, Snail was up-regulated upon HuR down-regulation can cause the expression of EMT-related proteins to be reversed. The radiation sensitivity of esophageal cancer was significantly reduced after Snail was up-regulated, and the radiation sensitivity caused by the down-regulation of HuR was much lower in cells with the up-regulation of Snail than those without Snail overexpression.

Studies have found that HuR participates in the regulation of Snail’s stability. Dong et al. found that HuR enhances the stability of Snail mRNA, and further enhances cell migration ability by inhibiting the expression of E-cadherin (18). HuR enhances the stability of Snail mRNA, thereby enhancing cell migration ability by inhibiting the expression of E-cadherin. Phosphorylated UGDH interacts with HuR to weaken the UDP-glucose-mediated inhibition of HuR binding to SNAI1 mRNA, thereby enhancing the stability of SNAI1 mRNA, increasing the epithelial-mesenchymal transition of lung cancer cells, and the migration of tumour cells and lung cancer metastasis (19). HuR binds to the 3’-UTR of Snail mRNA to stabilize Snail mRNA and increase the expression of Snail protein, leading to the formation of EMT, metastasis and stem cell-like cancer cells (CSC) in pancreatic cancer cells to promote the progression of pancreatic cancer (14). HuR can recognize AU-rich elements in Snail-encoded mRNA, thereby regulating Snail translation. The loss of HuR translocation induced by the tumor suppressor gene Scribble mediates the accumulation of Snail by activating the p38 MAPK pathway, leading to tumor drug resistance (20).

Snail-regulated E-cadherin expression plays an important role in the development of esophageal cancer (21–23). Guo et al. found that there is a positive correlation between the expression of EIF3H and Snail in esophageal cancer tissues. In esophageal cancer, EIF3H interacts and stabilizes Snail through deubiquitination. And EIF3H can promote the Snail-mediated EMT process of esophageal cancer (24). In addition, a variety of genes such as phospholipase C epsilon (PLCE1) (25), ubiquitin specific peptidase 26 (USP26) (26), ovarian tumor domain-containing ubiquitin aldehyde binding protein 1 (OTUB1) (27), Deubiquitinating enzyme PSMD14 (28, 29), polycomb chromobox proteins 8 (CBX8) (30), glioma-associated oncogene homolog 1(GLI1) (31), Notch-1 (32), SPSB3 (33) and T−cell immunoglobulin and mucin domain−containing protein−3 (TIM−3) (34) can participate in the progression of esophageal cancer by regulating the expression or stability of Snail. What’s more, non-coding RNA also participates in the progression of esophageal cancer by regulating the expression of Snail. Li et al. found that long non-coding RNA MEG3 (35) in esophageal cancer can inhibit the occurrence of EMT in esophageal cancer by inhibiting the PSAT1-dependent GSK-3β/Snail signaling pathway. Studies have found that MicroRNA-153 (36) and MicroRNA-30c (37) can inhibit the progression of esophageal cancer by regulating Snail. Moreover, Snail is related to the prognosis and overall survival rate of patients with esophageal cancer. Xu et al. found that Snail is an independent risk factor for the prognosis of patients with esophageal cancer. The combination of Snail and Twist can significantly affect the overall survival rate of patients with esophageal cancer (38). It has also been reported that Snail is involved in the regulation of radiosensitivity in esophageal cancer. Yang et al. found that knockdown of FAM83D inhibits the occurrence of EMT in human esophageal cancer cells through the Akt/GSK-3β/Snail signaling pathway, thereby enhancing the radiosensitivity of esophageal cancer (39).

Thus, HuR can affect the radiosensitivity of esophageal cancer by regulating the stability of the EMT-related protein Snail. HuR/Snail/EMT signal axis may be a potential target to enhance the radiosensitivity of esophageal cancer.

## Data Availability Statement

The original contributions presented in the study are included in the article/**Supplementary Material**. Further inquiries can be directed to the corresponding authors.

## Ethics Statement

The animal study was reviewed and approved by Soochow University. Written informed consent was obtained from the owners for the participation of their animals in this study.

## Author Contributions

XX and QW conceived and coordinated the study, designed the experiments. YH, QLi and KY performed and analyzed the experiments, and drafted the manuscript. CY and QLe carried out the data collection, analysis, and GW and XX revised the manuscript. All authors reviewed the results and approved the final version of the manuscript.

## Funding

This study was supported by the National Natural Science Foundation of China [Grant Number 81703022], Jiangsu Province Key Youth Talents Project [Grant Number QNRC2016262], Gusu Health Talents Training Project [Grant Number GSWS2019078], the guiding project of Jiangsu Provincial Health Committee [Grant Number Z2021077], and the project of Taicang Science and Technology Bureau (TC2018JCYL20). Changzhou Municipal Health Commission Major Science and Technology (ZD202104).

## Conflict of Interest

The authors declare that the research was conducted in the absence of any commercial or financial relationships that could be construed as a potential conflict of interest.

## Publisher’s Note

All claims expressed in this article are solely those of the authors and do not necessarily represent those of their affiliated organizations, or those of the publisher, the editors and the reviewers. Any product that may be evaluated in this article, or claim that may be made by its manufacturer, is not guaranteed or endorsed by the publisher.
