# Supplementary material for: HuR Affects the Radiosensitivity of Esophageal Cancer by Regulating the EMT-Related Protein Snail
Source: Front Oncol. 2022 May 19;12:883444. doi: 10.3389/fonc.2022.883444 (PMC9160430; doi:10.3389/fonc.2022.883444)
Supplement: Supplementary file 1 [file DataSheet_1.zip › Raw data.docx]

https://www.jianguoyun.com/p/DdMWHaoQls2_Chj6sroEIAA
