# Supplementary figures and images for: HuR Affects the Radiosensitivity of Esophageal Cancer by Regulating the EMT-Related Protein Snail
Source: Front Oncol. 2022 May 19;12:883444. doi: 10.3389/fonc.2022.883444 (PMC9160430; doi:10.3389/fonc.2022.883444)

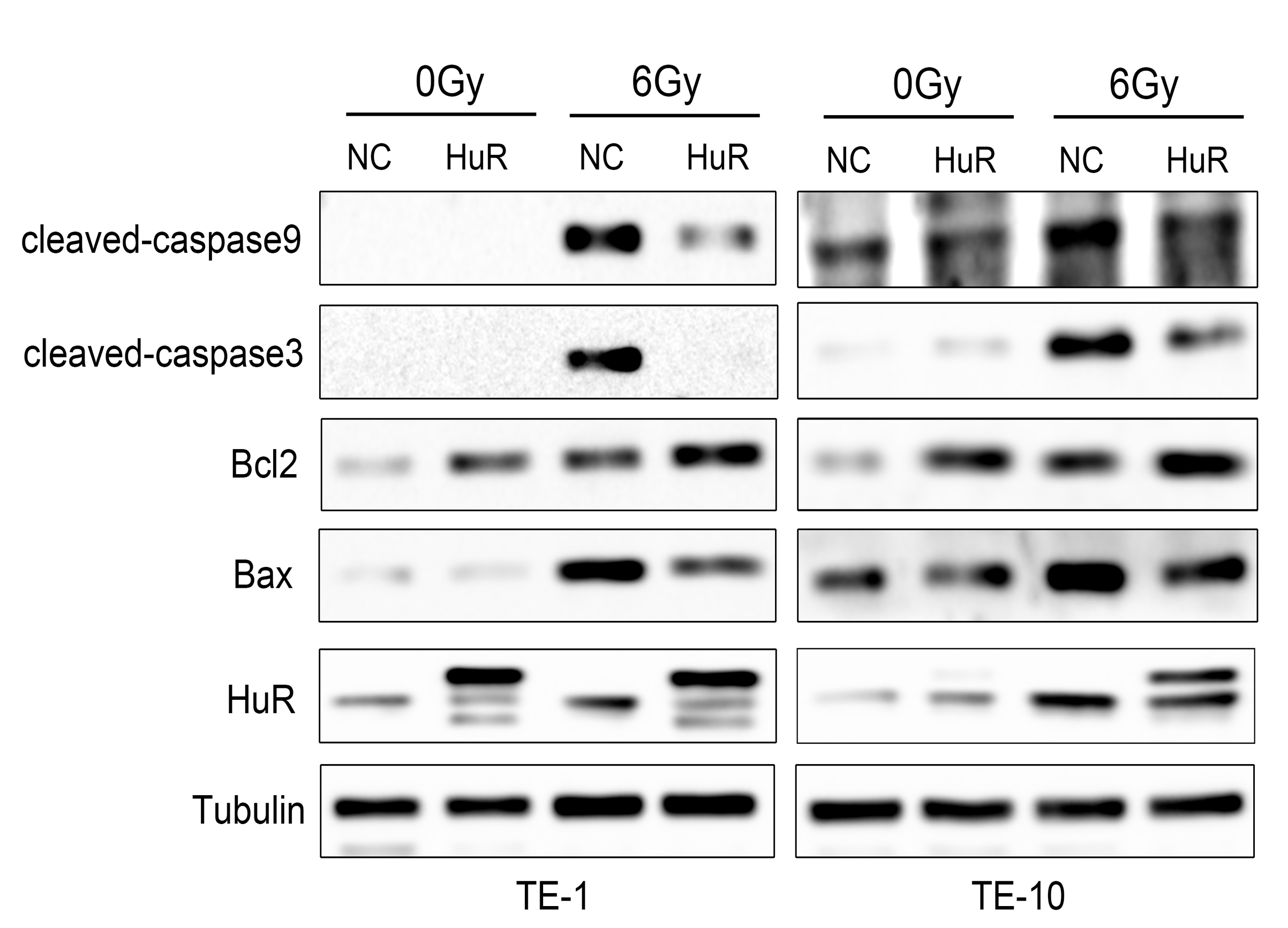

Supplement: Supplementary file 2 [file Image_1.tif]
